# Effects of Treatment and Weather Variables on Nocturnal Enuresis: Long-Term Analysis Using the Classification and Regression Trees Model

**DOI:** 10.7759/cureus.98841

**Published:** 2025-12-09

**Authors:** Yuta Onuki, Teruo Miyano, Yuma Iwanaka, Takahiro Ono, Shota Endo, Chisato Oyake, Yoshitaka Watanabe, Masaki Fuyama, Tsuneki Watanabe, Tomoko O Morita, Takahiro Arai, Hirokazu Ikeda

**Affiliations:** 1 Pediatrics, Showa Medical University Northern Yokohama Hospital, Yokohama, JPN; 2 Statistics, Japan Data Science Research Institute, Tokyo, JPN; 3 Pediatrics, Showa Medical University Fujigaoka Hospital, Yokohama, JPN

**Keywords:** cart model, long-term impact, nocturnal enuresis, treatment variables, weather variables

## Abstract

Objective: Nocturnal enuresis (NE), also known as bedwetting, is a common urological condition in children that often requires extended treatment. Although numerous studies have examined NE risk factors, the long-term effects of combined clinical and external factors, such as weather variables, remain unexplored. This study aimed to explore composite risk factors for NE and evaluate their long-term effects.

Methods: This was a prospective observational study with unbalanced panel data analyzed using the classification and regression trees (CART) model. The study was conducted in Japan, with clinical and meteorological data collected over 14 months. Nineteen pediatric patients aged 6-15 years diagnosed with NE based on International Pediatric Continence Society guidelines were included. In total, 3194 daily records from 19 participants (after excluding 1 due to excessive missing values) were analyzed. The primary outcome was the presence of incontinence. Factors evaluated included demographic variables, five treatment modalities (vasopressin, solifenacin succinate, vibegron, alarm therapy, and osmotic laxatives), and six meteorological factors (daily average ambient temperature, temperature difference, precipitation, sunshine hours, atmospheric pressure, and humidity).

Results: Risk factor analysis incorporating meteorological variables extracted multiple factors potentially contributing to nocturia in over 60% of patients. These included a history of desmopressin treatment without vibegron, fewer than 203 treatment days, absence of alarm therapy, and specific months (January, August, November, and December). Additionally, the presence of more than 82.5 treatment days and a daily average ambient temperature below 13.45°C were contributing factors. Meteorological variables, particularly daily average ambient temperature and calendar month, were selected as composite factors for NE risk via the CART model. Notably, vibegron was proposed to be more effective in reducing NE risk compared with other treatments.

Conclusions: Our findings suggest that effective long-term management of NE may require individualized treatment plans that account for clinical and weather-related variables. Among weather-related variables, consideration of seasonal and environmental factors may be especially important in managing NE.

## Introduction

Nocturnal enuresis (NE) is a disorder characterized by urination during nighttime sleep at least once a month after the age of five. NE is a common pediatric condition, affecting 15% of 6-year-olds and 6.4% of all children [1]. Although NE is not associated with physical symptoms, such as pain or fatigue, it markedly impacts a child’s mental well-being, contributing to low self-esteem, poor self-image, and a decline in school life quality and friendships [2, 3]. NE also reduces the overall quality of life for both affected children and their parents [4]. As a complex disorder, NE involves multiple factors, including increased urine production, urinary muscle overactivity, and sleep disturbances, with disorders of the autonomic nervous system believed to contribute to its onset [5-11]. Although several studies have explored the correlation between autonomic neuropathy and weather, the relationship between NE and weather remains unclear [12].

Standard treatments for NE include behavioral, medication, and alarm therapies [13, 14], with therapy choice tailored to the individual needs of the child and their family. However, treatment effectiveness varies markedly, and many cases remain unresponsive. Additionally, there is considerable variation in treatment duration, ranging from several weeks to years. Furthermore, NE recurrence is common, and many cases require ongoing follow-up and support. Despite the need for long-term treatment, few studies have investigated the actual long-term treatment practices. Therefore, this study aimed to identify combined risk factors for NE, including the influence of weather variables, and assess their long-term impact on the condition.

## Materials and methods

This study was approved by the Ethics Committee of Showa Medical University Hospital (approval number: 22-161-B), and written informed consent was obtained from all participants and their parents. This study established participant inclusion and exclusion criteria (Table 1).

**Table 1:** Participant Inclusion and Exclusion Criteria This study was conducted only on patients who met all of the above criteria.

Sr. No.	Inclusion and Exclusion Criteria
	Inclusion criteria
1	Intermittent urinary incontinence during sleep in children aged >5 years
2	Bedwetting at least once a month for ≥3 months
3	Primary monosymptomatic nocturnal enuresis
4	No missing data
	Exclusion criteria
1	Urinary tract infection within the preceding 3 months
2	Diabetes mellitus
3	History of renal disease, hypertension or genitourinary abnormalities, neurological disease, or psychological disease

**Figure 1:**
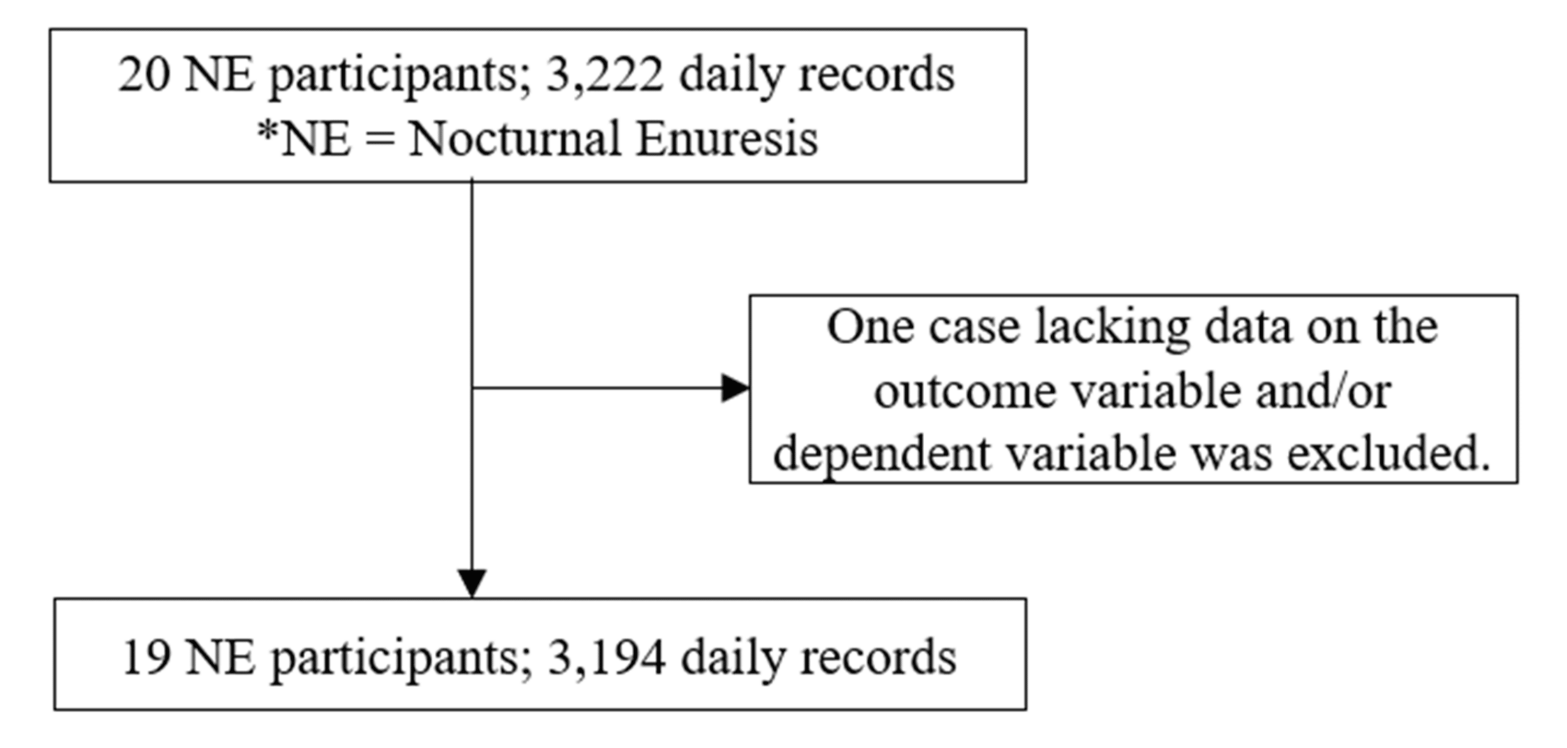
Flow diagrams Flowchart for this study

The study included pediatric patients aged 6-15 years diagnosed with NE based on International Paediatric Continence Society guidelines [13, 14]. The study period spanned from December 15, 2022, to February 1, 2024. Daily records of bedwetting occurrence were collected, resulting in 3,222 daily records. One participant with incomplete data on the outcome variable and/or dependent variable was excluded. Because the study also aimed to observe treatment interactions, we concluded that this case did not meet the criteria for inclusion as an observational participant. Consequently, 3,194 daily records from 19 individuals were included in the analysis (Figure 1).

This study utilized daily records for analysis to evaluate the impact of weather variables. Treatment duration varied widely, ranging from 1 day to 1,469 days, which allowed for the assessment of long-term treatment effects. The dataset included seven baseline variables, namely sex, age, residential region, height, weight, record date, and NE status (presence of incontinence). Five treatment dummy variables were included, namely desmopressin, solifenacin succinate, alarm therapy, and osmotic laxatives, which are recognized in the nocturnal enuresis guidelines, along with vibegron, which is not currently listed. Additionally, six major weather variables were extracted from Japan Meteorological Agency data [15] and included in the analysis: daily average ambient temperature, daily ambient temperature difference, total precipitation, sunshine hours, average atmospheric pressure, and average humidity. Meteorological data were matched with clinical records to create pooled data.

Given that the effects of weather variables and treatment duration on NE risk are nonlinear, and specific changes within a range can markedly affect NE outcomes, the classification and regression trees (CART) model was employed to account for the nonlinearity and complex patterns within the data. The CART model was adopted as the analytical method because it effectively ranks the importance of variables and identifies thresholds among multivariate causal analysis methods. Statistical analyses were performed using R version 4.3.3 (R Core Team). For the CART model analysis, the “rpart” function from the “rpart” package was used. Initially, the complexity parameter (cp) was set at 0.0001 to generate the largest possible tree. Cross-validation error (xerror) was then examined for tree pruning, and the final model was established using the cp value corresponding to the minimum xerror. Consequently, the final parameters were set to minbucket = 32 and cp = 0.0064.

## Results

Nineteen patients with NE were enrolled, and patient characteristics are provided in Tables 2-3.

**Table 2:** Patient characteristics Patient background and treatment details for participants in this study.

Characteristics		Overall (n = 19)
Age		
	Mean ± SD	9.58 ± 2.48
	Median	9
	Range	(6-15)
Sex		
	Male	12
	Female	7
Height		
	Mean ± SD	128.2 ± 11.1
	Median	127.2
	Range	111.0 - 147.7
Weight		
	Mean ± SD	27.2 ± 6.7
	Median	27.6
	Range	17.5 - 40.0
Treatment		
	Vasopressin (DDAVP)	11
	Solifenacin succinate	9
	Vibegron	6
	Alarm therapy	10
	Osmotic laxatives	4

**Table 3:** Treatment status for enuresis by case (days) Treatment for each patient

No.	Presence or Absence of Bedwetting	Number of Days of Observation	Presence or Absence of Treatment	DDAVP Used	Alarm Used	Solifenacin Used	Vibegron Used	Osmotic Laxative Used
1	24	223	183	0	0	223	182	0
2	55	141	141	0	0	141	141	0
3	49	315	315	0	0	292	256	311
4	263	317	317	317	0	218	0	98
5	43	260	260	180	260	180	0	0
6	24	78	78	0	78	0	0	78
7	28	245	245	0	237	0	245	0
8	24	161	161	0	0	0	161	0
9	78	80	80	80	80	0	80	0
10	81	273	273	273	273	273	0	0
11	28	98	90	0	90	0	0	0
12	61	154	154	154	154	154	0	0
13	9	134	134	109	64	64	0	0
14	23	133	77	77	0	0	0	0
15	143	154	98	98	98	98	0	0
16	6	203	203	203	0	203	0	0
17	12	64	64	64	0	28	0	64
18	18	75	64	64	0	0	0	0
19	64	86	56	0	56	0	0	0
Total	1033	3194	2993	1619	1390	1874	1065	551

**Figure 2:**
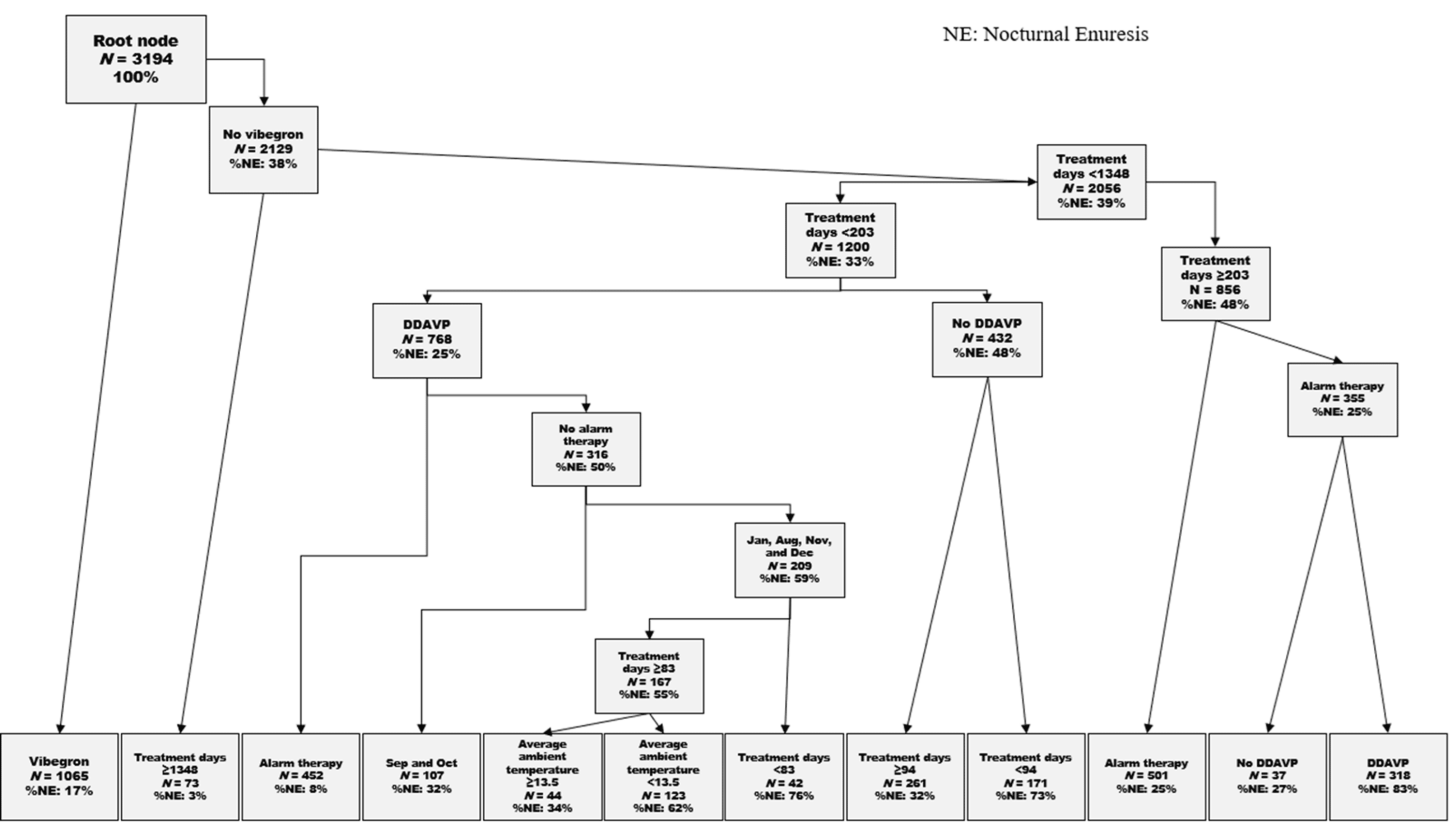
CART analysis Decision tree derived from this study. DDAVP: desamino-d-arginine vasopressin; CART: classification and regression trees

CART analysis, incorporating meteorological variables, selected several combined factors contributing to bedwetting in more than 60% of patients: a history of desmopressin use, no history of vibegron use, treatment duration of less than 203 days, a history of alarm therapy, calendar months (January, August, November, and December), treatment duration of ≥83 days, and average daily ambient temperature below 13.5°C (Figure 2).

With respect to vibegron, the risk of NE was 17% in the group with a history of vibegron treatment, whereas it was higher at 38% in the group without vibegron treatment. For desmopressin and alarm therapy, the risk of NE was significantly greater in the groups without a treatment history compared to those with a treatment history: 48% versus 25% for desmopressin, and 50% versus 8% for alarm therapy, respectively. Analysis by treatment duration showed that NE risk increased significantly to 48% with treatment duration of 203 days or more compared with 33% for less than 203 days, and 76% for less than 82.5 days compared with 55% for 82.5 days or more. Additionally, the risk of NE improved to 3% when treatment duration reached 1,348 days or more. For meteorological variables, the risk of NE was significantly higher at 62% when the mean ambient temperature was below 13.5°C compared to 34% at 13.5°C or higher. Notably, meteorological variables were selected as combined risk factors alongside pharmacological and behavioral therapies. Furthermore, vibegron, a β3-adrenergic receptor agonist, was also represented as a combined factor that reduces the risk of NE. Regarding treatment duration, variations in NE risk were suggested around 1,348, 203, and 83 days.

## Discussion

This study is the first to suggest that a meteorological factor, namely the daily average ambient temperature, influences the onset of NE. The key findings are as follows: (1) even when using desmopressin or alarm therapy, which are first-line treatments for NE, the risk of urinary incontinence increases as the daily average ambient temperature decreases; (2) although not explicitly recommended in current NE treatment guidelines, vibegron, a β3-adrenergic receptor agonist, was suggested to potentially contribute to reducing NE risk.

Previous studies by Tas et al. and Murillo et al. have shown that the incidence of NE episodes is significantly higher in winter than in summer, with increased failure rates of desmopressin treatment during winter. Our findings support this, suggesting that seasonal factors-specifically January, August, November, and December-increase the risk of NE-related urinary incontinence. Notably, January, November, and December correspond to winter months in Japan, characterized by particularly low ambient temperatures, consistent with prior reports (Table 4).

**Table 4:** Monthly ambient temperature (℃) in Tokyo

Year	January	February	March	April	May	June	July	August	September	October	November	December
2000	7.6	6	9.4	14.5	19.8	22.5	27.7	28.3	25.6	18.8	13.3	8.8
2001	4.9	6.6	9.8	15.7	19.5	23.1	28.5	26.4	23.2	18.7	13.1	8.4
2002	7.4	7.9	12.2	16.1	18.4	21.6	28	28	23.1	19	11.6	7.2
2003	5.5	6.4	8.7	15.1	18.8	23.2	22.8	26	24.2	17.8	14.4	9.2
2004	6.3	8.5	9.8	16.4	19.6	23.7	28.5	27.2	25.1	17.5	15.6	9.9
2005	6.1	6.2	9	15.1	17.7	23.2	25.6	28.1	24.7	19.2	13.3	6.4
2006	5.1	6.7	9.8	13.6	19	22.5	25.6	27.5	23.5	19.5	14.4	9.5
2007	7.6	8.6	10.8	13.7	19.8	23.2	24.4	29	25.2	19	13.3	9
2008	5.9	5.5	10.7	14.7	18.5	21.3	27	26.8	24.4	19.4	13.1	9.8
2009	6.8	7.8	10	15.7	20.1	22.5	26.3	26.6	23	19	13.5	9
2010	7	6.5	9.1	12.4	19	23.6	28	29.6	25.1	18.9	13.5	9.9
2011	5.1	7	8.1	14.5	18.5	22.8	27.3	27.5	25.1	19.5	14.9	7.5
2012	4.8	5.4	8.8	14.5	19.6	21.4	26.4	29.1	26.2	19.4	12.7	7.3
2013	5.5	6.2	12.1	15.2	19.8	22.9	27.3	29.2	25.2	19.8	13.5	8.3
2014	6.3	5.9	10.4	15	20.3	23.4	26.8	27.7	23.2	19.1	14.2	6.7
2015	5.8	5.7	10.3	14.5	21.1	22.1	26.2	26.7	22.6	18.4	13.9	9.3
2016	6.1	7.2	10.1	15.4	20.2	22.4	25.4	27.1	24.4	18.7	11.4	8.9
2017	5.8	6.9	8.5	14.7	20	22	27.3	26.4	22.8	16.8	11.9	6.6
2018	4.7	5.4	11.5	17	19.8	22.4	28.3	28.1	22.9	19.1	14	8.3
2019	5.6	7.2	10.6	13.6	20	21.8	24.1	28.4	25.1	19.4	13.1	8.5
2020	7.1	8.3	10.7	12.8	19.5	23.2	24.3	29.1	24.2	17.5	14	7.7
2021	5.4	8.5	12.8	15.1	19.6	22.7	25.9	27.4	22.3	18.2	13.7	7.9
2022	4.9	5.2	10.9	15.3	18.8	23	27.4	27.5	24.4	17.2	14.5	7.5
2023	5.7	7.3	12.9	16.3	19	23.2	28.7	29.2	26.7	18.9	14.4	9.4
Average ambient temperature (℃)	6.0	6.8	10.3	14.9	19.4	22.7	26.6	27.8	24.3	18.7	13.6	8.4

In contrast, August represents the summer season with the highest ambient temperatures; however, increased fluid intake during this month may disrupt fluid balance and elevate nocturnal urination risk, which likely explains its selection as a risk factor.

Regarding treatment duration, improvements were observed before 83 days and after 1,348 days from treatment initiation, whereas NE risk increased with treatment durations shorter than 203 days. Improvement before 83 days likely reflects the initial therapeutic response. The risk reduction after 1,348 days likely corresponds to the maturation of bladder function with age. Conversely, worsening risk after 203 days may be related to decreased motivation over prolonged treatment periods and seasonal temperature fluctuations. Although the treatment duration thresholds identified in this study serve as reference values, our long-term follow-up revealed that NE symptoms follow a nonlinear course characterized by cycles of exacerbation and remission, ultimately resulting in improvement.

NE guidelines recommend desmopressin and alarm therapy as first-line treatments and suggest anticholinergic agents and tricyclic antidepressants if these are ineffective [13, 14]. The CART analysis in this study confirmed that the therapies recommended in the guidelines reduce NE risk. Notably, vibegron was also suggested to potentially be effective for NE. Vibegron works by stimulating β3 receptors in the bladder smooth muscle [16], leading to muscle relaxation and improved urinary retention. Conversely, anticholinergic drugs inhibit excessive bladder contractions, in contrast to β3 receptor agonists, which relax the bladder, enhancing its ability to hold urine [17]. Previous clinical trials in adult women with an overactive bladder have shown the efficacy of β3 agonists compared to muscarinic receptor antagonists. For example, Vecchioli et al. reported that mirabegron (a β3 receptor agonist) significantly reduced post-void residual urine volume compared with solifenacin (a muscarinic antagonist) in such adults [17]. In contrast, the results of this study merely suggest the potential effectiveness of β3 receptor agonists for NE, and comparative trials to confirm their efficacy for this condition are awaited.

This study has several limitations. First, the small sample size and focus on a specific geographic region may limit the generalizability of the findings. Additionally, the complex interactions among weather variables such as barometric pressure, humidity, and sunlight hours require further investigation to fully understand their effects. A multifaceted approach to NE research is essential, taking into account not only physiological and psychological factors but also the influence of patients’ lifestyles. Furthermore, as this study is exploratory, confirmatory trials with larger sample sizes are needed to validate the efficacy of vibegron and the threshold values for outdoor temperature selected as composite factors.

Our study is the first to report the influence of an external environmental factor, namely the daily average ambient temperature, on NE symptoms, particularly bedwetting. Additionally, the risk of NE was shown to vary nonlinearly with changes in weather, treatment efficacy, and patient motivation. These findings underscore the importance of clinical management strategies that consider seasonality and environmental factors, suggesting practical recommendations such as advising families to maintain warm indoor temperatures during colder seasons, which may effectively reduce bedwetting incidence.

Study strengths and limitations

Strengths

To our knowledge, this is the first study to evaluate the impact of daily average ambient temperature on NE development using long-term treatment data. The CART model highlighted daily average ambient temperature, alongside clinical factors, as a key combined factor associated with NE risk.

Limitations

The study’s small sample size may limit the generalizability of the findings. In the analysis, daily records were used to examine the association between daily meteorological factors and NE; however, these records were based on individual cases and cannot be considered fully independent. Although vibegron, a β3 receptor agonist not currently listed in NE treatment guidelines, was suggested to reduce NE risk, further validation is required. Currently, trials comparing vibegron and muscarinic (M3) receptor antagonists are lacking. This study is exploratory, investigating whether meteorological variables are associated with NE risk. Therefore, further validation of the CART-derived cutoff values is required.

## Conclusions

This study proposes potential combined factors, including meteorological variables such as daily average ambient temperature, that influence the risk of urinary incontinence. The findings suggest that seasonal variations in temperature should be considered in the risk assessment of NE. Additionally, the potential efficacy of β3-adrenergic receptor agonists for NE was indicated, warranting future comparative trials. These results contribute to improved risk prediction and the development of more effective treatment strategies for NE.
